# Association of pro-inflammatory and anti-inflammatory cytokine polymorphisms with COVID-19 severity in unvaccinated patients

**DOI:** 10.3389/fimmu.2025.1641285

**Published:** 2025-08-13

**Authors:** Laura E. Martínez-Gómez, Carla I. Oropeza-Vélez, Maylin Almonte-Becerril, Leslie Chavez-Galan, Carlos Martinez-Armenta, Rosa P. Vidal-Vázquez, Juan P. Ramírez-Hinojosa, Paola Vázquez-Cárdenas, Diana Gómez-Martín, Gilberto Vargas-Alarcón, José M. Rodríguez-Pérez, Lucero A. Ramón-Luing, Julio Flores-Gonzalez, José G. Carrasco, Ivette Cruz-Bautista, Mónica M. Mata-Miranda, Gustavo J. Vázquez-Zapién, Adriana Martínez-Cuazitl, Nancy M. Parra-Torres, Felipe de J. Martínez-Ruiz, Dulce M. Zayago-Angeles, Ma. Luisa Ordoñez-Sánchez, Yayoi Segura-Kato, Carlos Suarez-Ahedo, Jessel Olea-Torres, Brígida Herrera-López, Carlos Pineda, Gabriela A. Martínez-Nava, Alberto G. López-Reyes

**Affiliations:** ^1^ Laboratorio de Gerociencias, Dirección General, Departamento de Reconstrucción Articular, Instituto Nacional de Rehabilitación Luis Guillermo Ibarra Ibarra, Secretaría de Salud, Mexico City, Mexico; ^2^ Servicio de Medicina Interna de Hospital Petróleos Mexicanos PEMEX, Mexico City, Mexico; ^3^ Dirección Ejecutiva de Investigación y Estudios Avanzados (DEIEA), Universidad de la Salud (UNISA), Mexico City, Mexico; ^4^ Laboratory of Integrative Immunology, Instituto Nacional de Enfermedades Respiratorias Ismael Cosio Villegas, Mexico City, Mexico; ^5^ Centro de Innovación Médica Aplicada, Hospital General Dr. Manuel Gea González, Mexico City, Mexico; ^6^ Department of Immunology and Rheumatology, Instituto Nacional de Ciencias Médicas y Nutrición Salvador Zubirán, Secretaría de Salud, Mexico City, Mexico; ^7^ Departamento de Biología Molecular y Endocrinología, Instituto Nacional de Cardiología Ignacio Chávez, Mexico City, Mexico; ^8^ Departamento de Endocrinología y Metabolismo, Instituto Nacional de Ciencias Médicas y Nutrición Salvador Zubirán, Mexico, City, Mexico; ^9^ Laboratorio de Biología Celular y Tisular, Laboratorio de Embriología, Escuela Médico Militar, Universidad del Ejército y Fuerza Aérea, Mexico, City, Mexico; ^10^ Nuevo Hospital General Delegación Regional Sur de la Ciudad de México ISSSTE, Mexico City, Mexico; ^11^ Unidad de Biología Molecular y Medicina Genómica, Instituto Nacional de Ciencias Médicas y Nutrición Salvador Zubirán, Mexico City, Mexico

**Keywords:** COVID-19, IL-6, IL-10, CCL-2, polymorphism, SARS-CoV-2

## Abstract

**Background:**

Cytokines and chemokines are essential for establishing an appropriate immune response to severe acute respiratory syndrome coronavirus-2 (SARS-CoV-2). Variations in the genes encoding cytokines and chemokines strongly influence the immune response to pathogenic challenges and disease outcomes. This study was conducted to investigate the associations between polymorphisms in the *TNF*-α, *IL-6*, *IL-8*, *IL-10*, and *CCL5* genes and COVID-19 severity.

**Methodology:**

We performed a cross-sectional study with a total of 627 unvaccinated COVID-19 patients were classified according to WHO disease severity. We evaluated the levels of IFN-α, IFN-γ, TNF-α, IL-1Ra, IL-2, IL-6, IL-7, IL-10, CCL2, CCL3, CXCL8, CXCL10 and GCSF in the serum and compared them among COVID-19 severity groups by Kruskal-Wallis test and stratified by polymorphism alleles. A logistic regression was performed to determine the association of the polymorphism and COVID-19 severity.

**Results:**

This study revealed a significant increase in IL-2, IL-6 and CCL-2 levels in the deceased group. However, the IL-10 levels were higher in the moderate group than in the mild group. Logistic regression analysis revealed that five polymorphisms were associated with a higher risk of severe COVID-19: the *TNF*-α (rs1800610) A allele (OR=1.50; 95% CI: 1.01–2.24); the *IL-6* (rs1800796) C allele (OR=1.64; 95% CI: 1.05–2.57); the *IL-10* (rs1800871) T allele (OR=1.94; 95% CI: 1.24–3.04) and (rs1800872) A allele (OR=1.87; 95% CI: 1.21–2.89); and the CCL5 (rs3817656) G allele (OR= 1.64; 95% CI: 1.02–2.65).

**Conclusion:**

Patients infected with SARS-CoV-2 who have the TNFα gene variant (rs1800629) are protected from developing COVID-19 moderate and severe outcomes, as well as from presenting low concentrations of some pro-inflammatory cytokines and chemokines. However, carriers of the IL-10 (rs1800872, rs1800871) and CCL-5 (rs2107538) gene variants were associated with patients who died from COVID-19. Of these, only the minor allele of CCL-5 was primarily associated with increased chemokines levels, as well as with some cytokines considered hallmarks of the cytokine storm.

## 1 Introduction

Severe acute respiratory syndrome coronavirus 2 (SARS-CoV-2) is the etiologic agent of coronavirus disease 2019 (COVID-19). This disease has a broad spectrum of clinical presentations, ranging from asymptomatic to severe outcomes such as acute respiratory distress syndrome (ARDS). Severe outcomes are often marked by hyper inflammation and elevated levels of proinflammatory cytokines such as IFN-α, IFN-γ and, IL-6, as well as chemokines like monocyte chemotactic protein-3 (MCP-1/CCL-2) (1–3).

In addition, different risk factors involved in outcome severity have also been described, including older age, male sex, obesity, and cardiovascular disease, among others. However, these risk factors do not explain all severe cases; genetic host variation might influence the clinical outcomes of patients with COVID-19 (4). Genetic variations may affect innate and adaptive immune responses (4, 5).

Polymorphisms in genes encoding pro-inflammatory cytokines may play a role in the pathogenesis of severe COVID-19 and could even influence their production. Single nucleotide polymorphisms (SNPs) in the *TNF-α*, *IL-6, IL-8, IL-10*, *CCL5* and *CXCL6* genes have been found to be associated with COVID-19 severity, suggesting that SNPs could help explain COVID-19 outcomes (2, 6–12).

In this context, the rs1800610 polymorphism in the TNF-α gene has been associated with several chronic diseases, including cancer, and the development of chronic obstructive pulmonary disease (COPD), as well as other infectious disease like hepatitis B virus (HBV) infection (13–15).

Recent studies have also highlighted strong associations between SNPs of IL6 (rs2069840) and IL10 (rs1800872) genes and the severity of COVID-19. Notably, the CC genotype of IL-10 appears to play a protective role against severe disease progression, although these SNPs may also indicate increased susceptibility to inflammatory conditions in general (2, 16).

A study reported by Alvarez et al., identified an association between SNP rs2107538 of CCL5 and bronchiolitis caused by respiratory syncytial virus (RSV), including the increased need for mechanical ventilation in affected patients (17). This SNPs has also been linked to human immunodeficiency virus (HIV) infection (18). Furthermore, a meta-analysis by Areeshi et al., demonstrated a significant association between rs2107538 and increased susceptibility to tuberculosis (TB) (19).

The aim of this work was to evaluate the associations of polymorphisms in the *TNF*-α, *IL-6*, *IL-8*, *IL-10*, *CCL5* and *CXCL6* genes with COVID-19 outcomes and with the serum levels of IFN-α, IFN-γ, TNF-α, IL-1Ra, IL-6, IL-7, IL-10, CCL2, CCL3, CXCL8, CXCL10 and GCSF.

## 2 Materials and methods

### 2.1 Setting and participants

From June 2020 to March 2021, nonprobability sampling was performed with unvaccinated patients in a multicenter cross-sectional study from different Mexican governmental health care institutions. This study was approved by the ethics committee of the Instituto Nacional de Rehabilitación Luis Guillermo Ibarra Ibarra (INR-LGII: 17/20). Informed consent was obtained from each participant. The inclusion criteria were as follows: either sex, age ≥18 years with clinical features of COVID-19, and a positive qRT–PCR test from a nasopharyngeal swab. The exclusion criteria were pregnancy, incomplete clinical records and individuals related to included participants. The participants were classified according to the WHO clinical progression scale and scored as follows: ambulatory mild disease (score of 1-3), hospitalized moderate disease (score of 4-5), severe disease (score of 6-9), and death (score of 10) (20).

### 2.2 Sample collection

Peripheral blood samples were collected at two days after hospital admission for COVID-19. One sample using EDTA anti-coagulant tubes was used for DNA extraction and other tube with clot activator/separator gel additives for serum collection. The latter was allowed to clot and centrifuged at 2,000 X G for 10 minutes to obtain the serum, which was stored at -80°C until further use. In the case of ambulatory patients, the samples were collected at the hospital´s triage, after the individual was confirmed to be positive by qRT-PCR test.

### 2.3 Selection and genotyping of SNPs

The polymorphisms of *TNF*-α (rs1800610 (+489 G/A), rs1800629 (-308 G/A), and rs3093664), *IL-6* (rs1800796 (-572 G/C) and rs10499563 (-6331 T/C)), *IL-8* (rs2227307), *IL-10* (rs1800872 (-819 C/T) and rs1800071 (-592 C/A)), *CXCL6* (rs4279174), and *CCL5* (rs2107538 and rs3817656) were selected based on their previous scientific evidence of associations with different diseases in any population that included independent genetic studies from 2003–2020.

Genomic DNA was isolated using a specialized commercial kit (QIAmp DNA Blood Mini Kit, Qiagen, Hilden, Germany). The quality of the DNA samples was evaluated by the 260/280 nm absorbance ratio, and 1% agarose gels were stained with SYBR^®^ Green (Invitrogen, CA, USA). The DNA concentration was subsequently quantified using a spectrophotometric method and adjusted to 20 ng/μl.

For genotyping, 10 ng/μl of genomic DNA was transferred into OpenArray plates, which previously contained the specific genotyping primers and probes, via the AccuFill system. Real-time PCR amplification was performed according to the supplier’s protocol via the OpenArray Platform through a Quant Studio 12K Flex System (Thermo Fisher Scientific, Waltham, MA, USA), and the results were analyzed using TaqMan Genotyper v1.6 software.

### 2.4 Soluble molecule evaluation

Serum IFN-α, IFN-γ, TNF-α, IL-1Ra, IL-2, IL-6, IL-7, IL-10, CCL2, CCL3, CXCL8, CXCL10 and GCSF levels were measured using a Human COVID-19 Cytokine Storm Panel 1 (14 plex) provided by Biolegend (Cat. No. 741088, Lot B377360) following the manufacturer´s instructions.

### 2.5 Statistical analysis

The normality of the variable distributions was evaluated. The Kruskal–Wallis test was used to compare nonparametric continuous variables among the studied groups, and the results are presented as the median and interquartile range (IQR). For the categorical variables, the chi-square test was performed. For all tests, a value of P <0.05 was considered to indicate statistical significance.

In addition, the linkage disequilibrium (LD) among all variants was determined using HaploView software V4.2. We tested 11 SNPs, Bonferroni test corrections was applied and correction of the P value was set at (α = 0.05/11) α = 0.004.

Logistic regression analysis adjusted for age, sex, hypertension status, type 2 diabetes status and obesity status was used to evaluate associations between genetic variants and the outcomes of COVID-19 patients. The final models were evaluated using the Hosmer–Lemeshow goodness-of-fit test. The analysis was performed using the STATA v.16 statistical package (StataCorp Texas, USA).

## 3 Results

### 3.1 Study population

In the present work, 627 subjects were included and stratified in 20% mild (n=123), 36% moderate (n=229), 26% severe (n=162) and 18% dead (n=113). **Table 1** shows the features and clinical information of the study population. The median age of the total population was 52 years (IQR 43-63), 63% were males, and the principal comorbidities were obesity in 31% (n=197), hypertension in 30% (n=188) and type 2 diabetes mellitus in 31% (n=193).

**Table 1:** Anthropometric and clinical characteristics of population study.

	Total 627 (100%)	Mild 123 WHO score 1-3 (20%)	Moderate 229 WHO score 4-5 (36%)	Severe 162 WHO score 6-9 (26%)	Dead 113 WHO score 10 (18%)	P value
Age (years) ^#^	52 (43-63)	40 (30-49)	52 (42-63)	52 (46-63)	63 (54-70)	**<0.001**
Sex Male	394 (63%)	59 (48%)	146 (64%)	116 (72%)	73 (65%)	**0.001**
Type 2 diabetes -yes	193 (31%)	14 (11%)	76 (33%)	54 (33%)	49 (43%)	**<0.001**
Obesity -yes	197 (31%)	18 (15%)	74 (32%)	67 (41%)	38 (34%)	**<0.001**
Hypertension -yes	188 (30%)	12 (10%)	67 (29%)	60 (37%)	49 (43%)	**<0.001**
D-Dimer (ng/mL) ^#^	295 (75-682)	245.5 (133.5-386.5)	266 (39-682)	459 (83-849)	322.7 (133-872)	**<0.001**
Ferritin (ng/mL)	445.6 (201.3-823.5)	121.1 (24-316.5)	415.5 (206.5-747.4)	572.2 (76.2-998.2)	692.7 (395-1213.7)	**<0.001**
LDH (U/L) ^#^	300.5 (207-436.5)	151 (121.5-194.5)	281 (221-381)	394 (280-482)	407 (322-488.4)	**<0.001**
C-Reactive Protein (mg/L) ^#^	18.3 (5.2-63.9)	2.8 (1.1-9.5)	30.7 (6.9-96.4)	19.1 (7.9-33.3)	23.2 (14.7-118.7)	**<0.001**
IFN α (pg/mL) ^#^	18 (10.8-33.3)	18.16 (13.48-25.47)	23 (15.8-46.2)	14.9 (6.1-33.3)	15.8 (5.6-25.5)	**<0.001**
IFN γ (pg/mL) ^#^	51.71 (38.8-90.9	44.4 (36.6-72.2)	61 (42.1-91)	60.9 (42.7-97.9)	48.7 (37.4-79.4)	**0.03**
TNFα (pg/mL) ^#^	59.8 (20.3-183.1)	28.8 (15.1-135.1)	173.7 (25.7-316.3)	91.3 (34.7-168.7)	42.6 (15-78.6)	**<0.001**
IL-1Ra (pg/mL) ^#^	215.9 (52.8-408.4)	37.7 (24.1-175.4)	176 (70.5-244.8)	365 (215.4-674)	336.7 (137.5-674.1)	**<0.001**
IL-2 (pg/mL) ^#^	106.6 (31.8-294.3)	28.2 (14.8-83.8)	109.8 (44.4-215.3)	135.1 (60.3-409.3)	367.5 (163.9-930.9)	**<0.001**
IL-6 (pg/mL) ^#^	106.6 (31.8-294.3)	28.9 (14.8-84.2)	109.8 (44.4-215.3)	135.0 (60.2-409.3)	362.3 (163.9-743.6)	**<0.001**
IL-7 (pg/mL) ^#^	127 (73.9-405.1)	70.6 (32.3-297.8)	404.7 (111.2-692.8)	140.7 (86.6-318.1)	109.4 (79.9-149.4)	**<0.001**
IL-10 (pg/mL) ^#^	40.4 (20.1-101.5)	33.2 (19.8-86.3)	65.5 (32.3-131.9)	33.5 (14.5-101.9)	40.5 (23.1-67.5)	**0.03**
CCL2 (pg/mL) ^#^	1160.3 (352.8-2339.7)	330.8 (192.9-1292.3)	1025.6 (356.9-2021.5)	1400 (755.5-2374.9)	2375.2 (1320.8-4296.1)	**<0.001**
CCL3 (pg/mL) ^#^	169.1 (27.2-363.5)	31.9 (0.5-241)	115.5 (41.9-264)	290.1 (106.4-465)	240.8 (42.6-442.8)	**<0.001**
CXCL8 (pg/mL) ^#^	179.3 (55.2-334.2)	42.6 (19.5-204.6)	256.8 (48.8-441.1)	207.5 (147.3-270.3)	202.6 (149.7-270.3)	**<0.001**
CXCL10 (pg/mL) ^#^	1238.1 (642.4-2153.2)	697.1 (332.6-1829.5)	1400.4 (1012.1-2567.9)	1353.3 (723.1-2117.9)	2003.3 (1083.2-2492.9)	**<0.001**
GCSF (pg/mL) ^#^	28.9 (16.7-57.7)	24.8 (18.1-51.4)	41.9 (25.5-64.7)	27.1 (12.31-61.4)	31.9 (17.9-49.3)	**0.08**

^#^Median (Interquartile Range). P-value obtained from Kruskal-Wallis Test. Values in bold denotes statistical significance.Values in bold denotes statistical significance with a P-value <0.05.

For clinical biomarkers, we observed statistical significance (*p*<0.001) among the groups, where ferritin, lactate dehydrogenase (LDH) and CRP levels tended to increase with the severity of the disease (**Table 1**).

### 3.2 Cytokine levels among severity COVID-19

Concerning IL-6 and CCL2, we observed a tendency toward higher levels (pg/mL) as the severity of the disease increased. Similarly, CXCL-10 showed the highest concentration in the deceased group, and IL-1Ra and CCL3 also presented the highest concentrations in the severe and deceased COVID-19 groups. Interestingly, the concentrations of IFN-α, IFN-γ, TNF-α, IL-2, IL-10, CXCL8 and GCSF were the highest in the moderate disease group (**Table 1**). We performed a Spearman correlation analysis with the WHO severity score and observed a moderate positive correlation with IL-6 (ρ=0.53), IL-1 (ρ=0.52) and CCL2 (ρ=0.43) (p ≤ 0.0001).

### 3.3 Allelic and genotypic frequencies and linkage disequilibrium

The allelic and genotypic frequencies are shown in **Supplementary Table S1**. We observed a significant difference in the distribution of the allele frequencies among the study groups for the *IL-6* rs1800796 (-572 G/C) and rs10499563 (-6331 T/C); *IL-10* rs1800871 (-819 T/C) and rs1800872 (-592 C/A); and *CCL5* rs3817656 polymorphisms (**Supplementary Table S1**). The LD was calculated for all SNPs; however, only rs1800871 (-819 T/C) and rs1800872 (-592 C/A) of the *IL-10* gene showed LD, with R^2^ values of 0.99 and D´=0.99, as did rs2107538 and rs3817656 of CCL5 (R^2^ values of 0.79 and D´=0.95).

### 3.4 Logistic regression analysis

**Figure 1:**
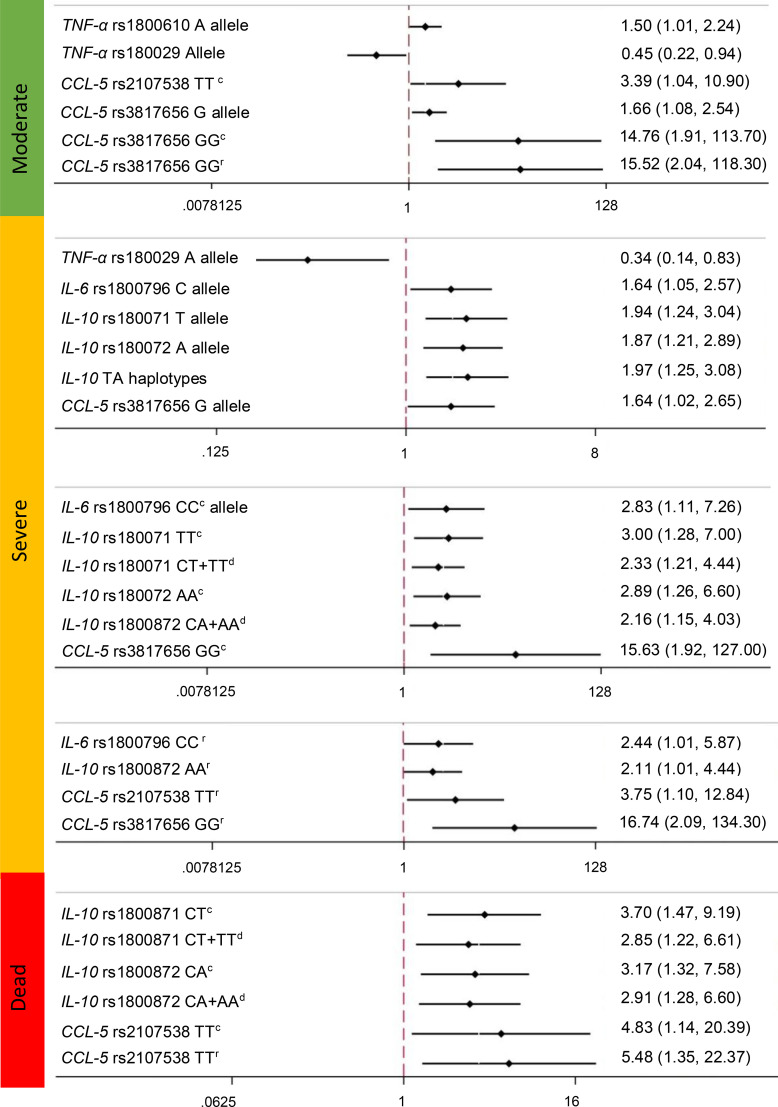
Association of the Polymorphisms of *TNFα* (rs1800610, rs1800629, rs3093664), *IL-6* (rs1800796, rs10499563), *IL-10* (rs1800872 and rs1800071), and *CCL5* (rs2107538, rs3817656) genes and COVID-19 outcomes. c, Codominant; d, dominant; r:recesive. OR (CI). Association statistically significant differences among groups. The models were adjusted by the main covariates: type two diabetes, obesity, hypertension, sex and age.

In the **Figure 1**, showed the association of the SNPs, among COVID-19 severity. For the TNF-α gene, we observed an association of the rs1800610 risk allele with moderate disease (OR=1.5, 95% CI= 1.01-2.24, p=0.04), as well as a protective association of the rs1800629 minor allele with moderate disease (OR=0.45, 95% CI95% CI= 0.22-0.94, p=0.03) and severe outcomes (OR=0.34, 95% CI95% CI=0.14-0.83, p=0.02) (**Supplementary Table S2**). In severe outcomes, for *IL-6* gene, we found an association for the rs1800796 (-572 G/C) C allele (OR of 1.64 (95% CI=1.05–2.57, *p*=0.03). Additionally, for the CC genotype of the codominant model, the OR was 2.83 (95% CI=1.11–7.26; *p*=0.03). In addition, for the CC of the recessive model, we found an OR of 2.44 (95% CI=1.01-5.87, *p*=0.04) (**Supplementary Table S3**). For rs1049953 (-6331T/C), we did not observe a significant association with any outcome.

Interestingly, for the *IL-10* gene rs1800871 (-819 C/T) polymorphism, we observed a significant association between the risk allele and severe outcomes with an OR of 1.94 (95% CI=1.24-3.04, p=0.004). The TT genotype of the codominant model had an OR=3.00 (95% CI=1.28-7.00, *p*=0.01), and the CT+TT genotypes of the dominant model had an OR=2.33 (95% CI 1.21-4.44, *p*=0.01) for severe outcomes. In addition, for the deceased outcome, we observed a significant association with CT in the codominant model (OR=3.7 95% CI=1.47-9.19, *p*=0.005) and CT+TT in the dominant model (OR=2.85 95% CI=1.22-6.61, *p*=0.02). For rs1800872 (-592 C/A), the A allele was associated with severe and deceased outcomes, with ORs of 1.87 (95% CI=1.21-2.89, *p*=0.004) and 1.73 (95% CI=1.01-2.9, *p*=0.04), respectively. The AA genotype of the codominant model had an OR of 2.89 (95% CI=1.26-6.60, *p*=0.01), the CA+AA genotype of the dominant model had an OR=2.16 (95% CI=1.15-4.03, *p*=0.02), and the AA genotype of the recessive model had an OR=2.11 (95% CI =1.01-4.44, *p*=0.04), all of which were associated with severe outcomes. In addition, we observed a significant association between the CA genotype of the codominant model and the CA+AA genotypes of the dominant model with a deceased outcome, with ORs of 3.17 (95% CI=1.32-7.58, *p*=0.009) and 2.91 (95% CI=1.28-6.60, *p*=0.01), respectively (**Figure 1**).

We performed logistic regression with the haplotypes and found a significant association between the CT haplotype and severe outcomes, with an OR=1.97 (95% CI=1.25-3.08, *p*=0.003) (**Supplementary Table S2**).

For the *CCL5* gene, the rs2107538 in the codominant model (AA genotype) was significantly associated with moderate (OR= 3.39 95% CI= 1.04-10.9, *p=*0.04) and deceased (OR= 4.83 95% CI= 1.14-20.39, *p=*0.03) outcomes. The recessive model (TT genotype) tended to increase the associations among the COVID-19 outcomes (**Supplementary Table S2**). Variant rs3817656, the G allele, was significantly associated with moderate (OR= 1.66 95% CI= 1.08-2.54, *p=*0.02) and severe (OR= 1.64 95% CI= 1.02-2.65, *p=*0.04) COVID-19 outcomes (**Supplementary Table S2**).

When we adjusted by Bonferroni correction, only the *IL-10* gene rs1800871 (-819 C/T) polymorphism conserved the significant association between the T allele and severe outcome, with an OR of 1.94 (95% CI=1.24-3.04, p=0.004), as well as the association seen for the A allele of the rs1800872 polymorphism with severe outcome (OR=1.87 (95% CI=1.21-2.89, *p*=0.004).

### 3.5 Cytokine levels and SNPs

We performed a sub-analysis to determine the relationships between the concentrations of the cytokines previously mentioned with the SNPs. In this analysis, the carriers of the risk allele of the *TNF-α* rs1800629 polymorphism presented lower levels of IL-1Ra, IL-6, CCL-2, and CXCL10 than did the carriers of the major allele (**Supplementary Table S3**).

Interestingly, we observed differences for the rs1049953 (-6331T/C) polymorphism of the *IL-6* gene concerning the level of *IFN-α*. In addition, we observed that carriers of the risk allele of this SNP had the highest serum concentrations of IL-6 and CCL-2 (**Supplementary Table S3**).

Similarly, for the *CCL-5* rs2107538 polymorphism, carriers of the A allele had the highest serum levels of the IL-2, IL-6, IL-7, IL-10, CCL-2, GCSF, and CXCL10 cytokines. For rs3817656, carriers of the risk allele presented increased serum levels of the cytokines IL-2, IL-6, IL-7, CCL-2, GCSF, and CXCL8. These differences were also found to be significant for the *CCL-5* haplotype AG, except the GCSF concentration, which did not reach significance (**Supplementary Table S3**).

## 4 Discussion

In COVID-19, severe outcomes have increased levels of pro-inflammatory and anti-inflammatory cytokines and chemokines, such as IL-6, TNF-α, IL-10, CCL2, CCL3, CXCL8, and CXCL10, among others (1, 3, 4). Since COVID-19 emerged, several studies across the world have reported some polymorphisms of different genes related to this disease (4, 5). SNPs in the *TNF-*α, *IL-6*, *IL-8*, *IL-10*, *CCL5* and *CXCL6* genes have been associated with COVID-19 outcomes and with serum cytokine and chemokine levels (3, 6).

We observed a trend toward increased CCL2 levels with the outcomes of the COVID-19 patients, as well as, of IL-1Ra and CCL3 levels being elevated in patients with severe disease. These observations are consistent to those reported by Torres-Ruiz et al. (2021), who reported a tendency for CCL2, CCL3, and other molecules to increase with disease severity (3, 6). Additionally, Dorgham et al. (2021) revealed increased levels of IFN-α and, TNF-α in patients who died from COVID-19, supporting the idea that inflammatory cytokines play a key role in disease progression (21, 22).

Several genome-wide association studies (GWASs) and candidate gene studies have identified SNPs associated with susceptibility to SARS-CoV-2 infection and severe COVID-19 (4, 5, 23).

TNF-α promotes detrimental tissue damage and gradual lung fibrosis, which results in pneumonia, pulmonary edema, and acute respiratory distress syndrome (21). In addition, the *TNF-α* gene is highly polymorphic, the rs1800610 is located in the first intron of the gene +489 (G/A), and rs1800629, which is located in the promoter region of the gene -308 (G/A). Our results revealed that only the rs1800610 A allele was significantly associated with the risk of a moderate COVID-19 outcome. This SNP in the Australian population was associated with the risk of developing breathing problems with bronchial exacerbation, suggesting that this SNP could contribute to disease progression. In addition, rs1800610 tends to increase cytokine concentrations in carriers of the A allele, and is related to prostate cancer and COPD development (7, 15). We were also able to demonstrate that the rs1800629 (-308 G/A) was found to be associated with protection against moderate and severe disease. The protective effect of rs1800629 (-308 G/A) is consistent with that reported by Heideri Nia et al., who showed that allele A decreases the risk of SARS‐CoV‐2 infection (24).

Interestingly, we identified a significant association between the A allele of the rs1800629 SNP with severe COVID-19. Due to the absence of AA genotype among individuals with mild, severe, and dead outcomes, standard genetic model analyses could not be performed. Nevertheless, rs1800629 has been previously reported to modulate the expression of key components within the inflammatory signaling cascade, which could influence the production of pro-inflammatory cytokines such as IL-6, TNF-α, IFN-γ, particularly in patients with metabolic syndrome (25, 26).

The role of IL-6 has been implicated in the regulation of genes and proteins involved in angiogenesis and immune cell recruitment (6). In addition to these roles, IL-6 exerts pleiotropic effects, contributing to a wide range of physiological and pathological processes, including inflammation, tumorigenesis, and immune modulation (8).

Genetic variations in the *IL-6* promoter region, particularly the SNPs rs1800796 (-572 G/C) and rs10499563 (-6331 T/C), have been associated with immunological regulation of hepatocellular carcinoma and other inflammatory pathologies. These SNPs contribute to the functional regulation of expression of this gene (2, 27). We found that the C allele of the (-572 G/C) rs1800796 polymorphism was associated with severe COVID-19. However, Falahi et al. reported no association between the (-572 G/C) rs1800796 polymorphism and COVID-19 severity in the Kurdish population (28). However, their logistic regression analyses were not adjusted for major comorbidities and were conducted on a relatively small sample size, which may limit the reliability of their findings. By contrast, our study benefits from a larger sample size and incorporates adjustments for key comorbid conditions, thereby enhancing the robustness and validity of the results.

Karcioglu et al. investigated the correlation between rs1800796 (-572 G/C) frequency and incidence and mortality of COVID-19 across 23 countries. Their analysis did not reveal a statistically significant correlation. However, it is important to note that the dataset included two studies from Mexico, one involving patients with hip fractures and another involving individuals infected with HBV, neither of which focused on COVID-19 outcomes (29). Our study included individuals with severe COVID-19, and revealed genetic association with disease severity, supported by robust statistical models, showing the genetic contribution to this disease.

IL-8 has effects through binding to cognate G-protein-coupled CXC chemokine receptors, CXCR1 and CXCR2, which activate a phosphorylation cascade to trigger chemotaxis and neutrophil activation as part of the inflammatory response. In COVID-19, the plasma IL-8 concentration is increased, suggesting a significant role in cytokine release syndrome, multiorgan dysfunction, respiratory failure and shock (9, 30). SNPs in the *IL-8* gene could influence the molecular mechanisms of COVID-19. Nevertheless, in our study, we did not find an association between rs2227307 and COVID-19 severity.

Previously, in Mexico, rs1800871 (-819 C/T) and rs1800872 (-592 C/A) were found not to be associated with COVID-19 severity. However, the sample size was small (193 subjects), which means that the statistical power was low (31). Abbood et al. (2023), in an Iranian population, reported that rs1800871 (-819 C/T) and r1800872 (-592 C/A) were related to COVID-19 mortality (32). Monroy-Muñiz et al. (2023) studied rs1800871 (-819 C/T) and r1800872 (-592 C/A) in a Mexican population and reported that the T and A alleles were associated with the risk of dengue infection (33). Our study reported that T and A alleles were associated to COVID-19 severity adjusted by Bonferroni test. The SNPs rs1800871 (-819 C/T) and rs1800872 (-592 C/A), of *IL-10* gene, are located in the 5´-flanking region (7). In autoimmune diseases, like ankylosing spondylitis, increased levels of IL-10 in the serum have been described. Ling et al. (2021) reported that IL-10 serum levels are greater in patients admitted to the ICU than in those who are not (34). It has been reported that rs1800872 (-592 C/A) can reduce negative promoter function, and therefore modify IL-10 transcription (31, 32).

CXCL6 is a chemokine characterized by four conserved cysteine residues close to the amino terminus CXC motif; In lung inflammation, CXCL6 stimulates inflammation by recruiting and activating immune cells. However, in our study we did not observe a significant association between rs4279174 of the *CXCL6* gene and COVID-19 severity. However, other variants in this gene may influence disease severity.

CCL5, also called RANTES, has been studied in many inflammatory diseases. Recently, the CCL5/CCR5 axis was reported to participate in the response to COVID-19, HIV and tuberculosis. Kouhpayeh et al. reported that the GA genotype in the dominant model of *CCL5* (rs2107538) was associated with an increased risk of pulmonary tuberculosis (12). Our results revealed that this SNP in the recessive model was associated with COVID-19 severity. However, Pati et al. reported that this variant was associated with protection against SARS-CoV-2 infection, but the mechanisms are unknown and were not adjusted by confounding factors such as sex or age, among others (35). However, our analysis was adjusting for sex, age, type 2 diabetes and hypertension, we also observed higher concentrations of cytokines in the presence of the risk alleles. The rs21075238 is located at the binding site for GATA binding protein 2 (GATA2), which is involved in the transcriptional regulation of proinflammatory cytokines therefore, CCL5 might participate in the COVID-19 inflammatory process (36, 37).

CCL-2 participates in multiple lung inflammatory diseases, and its major mechanism involves mediating the migration of mononuclear cells into the microvasculature and airways. This could explain the monocyte and lymphocyte influx observed in ARDS, asthma and bronchiolitis obliterans syndrome (BOS) (38). A recent report by Abers et al. suggested that CCL-2 was associated with mortality due to COVID-19 (38). Pius-Sadowska et al. reported that lower concentrations of CCL-2 mRNA but higher concentrations of the protein in plasma were correlated with more severe courses of COVID-19, suggesting that CCL-2, like other cytokines, could be considered new prognostic factors for severe COVID-19 (39).

In our study, when stratifying serum cytokine concentrations by alleles of the SNPs investigated, we observed that carriers of the A allele of rs1800629 of *TNF-α* gene showed the lowest levels of IL-1Ra, IL-6, CCL2, and CXCL10, whereas carriers of the G allele showed higher levels. Consistent with our findings Szkpu M et al., reported that the G/G genotype of rs1800629, showed higher IL-6 levels in elderly women with metabolic syndrome. These observations support the hypothesis that autoinflammatory mechanisms may contribute significantly to the pathophysiology and complications of the disease. Furthermore, the A allele has been associated to increased transcriptional activity (26).

**Figure 2:**
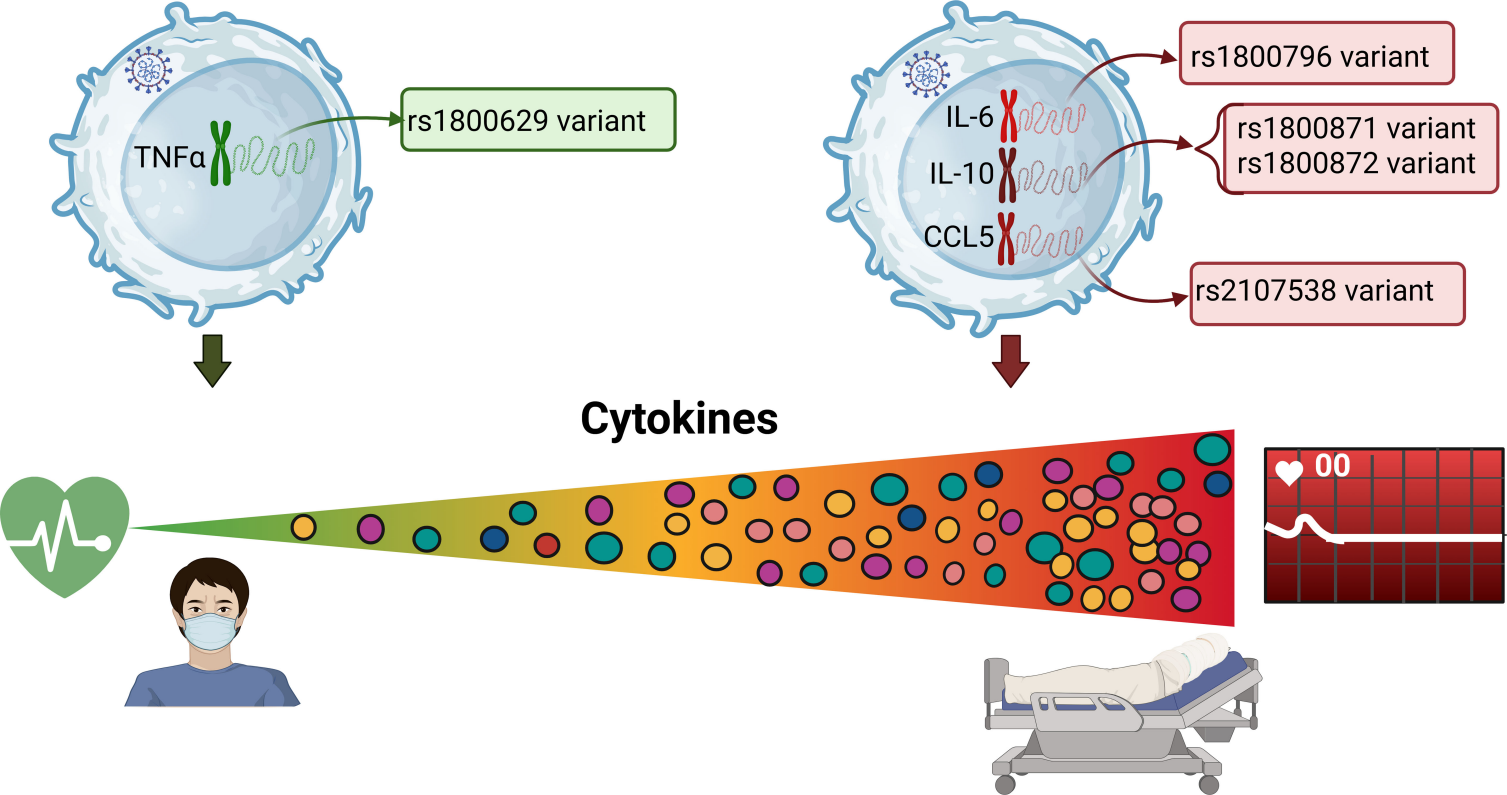
Association of the SNPs and cytokines, with COVID-19 severity.

Interestingly, we observed higher levels of IFN-α, IL-6 and CCL2 with the C allele of the rs10499563 (-6331 T/C) of *IL-6* gene. This SNPs is located near a distal regulatory sequence upstream of IL-6 expression via affecting the binding affinity of the transcription factor Oct-1 to its response element (**Figure 2**). Smith et al., demonstrated that this SNP is a significant predictor of increased IL-6 concentrations six hours after cardiac surgery. Other SNPs in close proximity to trs10499563 (-6331 T/C), such as rs18000795 and rs1800796 of *IL-6* gene, have also been linked to elevated serum IL-6 levels. In our study, carriers of the variant allele of rs1800796 showed increased IL-6 levels, although this association did not reach statistical significance (40–42). Additionally, we observed increased levels of IFN-α, and CCL2 in patients with the variant alleles of the rs10499563 (-6331 T/C). These SNPs could contribute to increased IL-6 levels, which can increase CCL2 production and promote immune cell recruitment. While the impact of these SNPs on IFN-α is less clear, IL-6 can influence the broader immune environment, potentially enhancing IFN-α responses in viral infections (6, 23).

We also observed elevated levels of IL2, IL-6, IL-10, CCL2, CXCL8, CXCL10 and GCSF in the T allele of the rs2107538 variant of *CCL5* gene. Silva et al, reported that rs217538 has a positive regulatory impact on CCL5 protein production in HIV-1 infection. Additionally, Zhernakova reported that several studies have shown that rs2107538, located in the first intron, can influence the transcriptional activity of CCL5, potentially contributing to susceptibility to autoimmune diseases. However, these findings have primarily been derived from case-control studies (18, 43).

Genetic variability among different populations also help us understand the diversity of disease course and susceptibility to its complications. It is possible that others variants not evaluated in this study are in LD and could influence cytokine levels.

In clinical practice, the tendency of certain cytokines to increase with the severity of COVID-19 is a significant concern. Among them, IL-6 has emerged as a key driver of inflammation. Targeting IL-6 with specific therapies has become a potentially life-saving strategy for patients with severe COVID-19. There are three main types of IL-6 inhibitors, typically monoclonal antibodies, including siltuximab, tocilizumab and sarilumab. These drugs have been approved by the Food and Drug Administration (FDA), for use in various conditions. Further pharmacogenetics studies are needed to determine whether specific IL-6 gene variants could serve as prognostic markers for patients response to these therapies in COVID-19 (44, 45).

This is the first comprehensive study evaluating genetic profile and cytokine levels in unvaccinated Mexican COVID-19 patients, adjusted for main comorbidities. However, the study has several limitations, including the possibility that patients with other underlying conditions may have compromised immune system, as well as genetic substructure may influence the observed associations, or that the findings may only be representative of central Mexican populations.

Further investigations of other SNPs in these genes or others implicated in the mechanism of COVID-19 severity are necessary for developing prevention actions and precision medical therapies. It should be noted that our results are only representative of the Mexican-Mestizo population.

This study highlights the associations of the IL-6 rs1800796 (-572 G/C), IL-10 rs1800871 (-819 C/T), rs1800872 (-592 C/A) and CCL5 rs2107538 polymorphisms with fatal COVID-19 outcomes and elevation of cytokines (CCL-2, IL-6, IL-10 and CXC-L10) plays a crucial role in the pathogenesis of COVID-19. However, Patients infected with SARS-CoV-2 who have the TNF-α gene variant (rs1800629) are protected from developing COVID-19 moderate and severe outcomes, as well as from presenting low concentrations of some pro-inflammatory cytokines and chemokines (IL-6, CCL2, and CXCL-10).

## Data Availability

The original contributions presented in the study are included in the article/**Supplementary Files**. Further inquiries can be directed to the corresponding authors.
