# Supplementary material for: Association of pro-inflammatory and anti-inflammatory cytokine polymorphisms with COVID-19 severity in unvaccinated patients
Source: Front Immunol. 2025 Aug 13;16:1641285. doi: 10.3389/fimmu.2025.1641285 (PMC12380819; doi:10.3389/fimmu.2025.1641285)
Supplement: Supplementary file 1 [file Table1.docx]

Supplementary Material

# Supplementary Tables

**Table S1 Allelic and Genotypes Frequencies in Mild, Moderate, Severe and Dead patients COVID-19**

|  | **Total 627 (100%)** | **Mild 123**  **(20%)** | **Moderate 229 (36%)** | **Severe 162 (26%)** | **Dead**  **113 (18%)** | P **value**^#^ |
| --- | --- | --- | --- | --- | --- | --- |
| TNF-a |  |  |  |  |  |  |
| rs1800610 (+489 G/A) |  |  |  |  |  |  |
| G | 864 (73%) | 175 (76%) | 307 (69%) | 224 (75%) | 158 (73%) | 0.19 |
| A | 326 (27%) | 55 (24%) | 137 (31%) | 76 (25%) | 58 (27%) |  |
| GG | 322(54%) | 70(61%) | 107(48%) | 84(56%) | 61(56%) | 0.37 |
| GA | 222(37%) | 35(30%) | 93(42%) | 56(37%) | 38(35%) |  |
| AA | 52(9%) | 10(9%) | 22(10%) | 10(7%) | 10(9%) |  |
| rs1800629  (-308 G/A) |  |  |  |  |  |  |
| G | 1144 (95%) | 209 (92%) | 421 (95%) | 307 (97%) | 207 (96%) | 0.06 |
| A | 60 (5%) | 19 (8%) | 21 (5%) | 11 (3%) | 9 (4%) |  |
| GG | 545(90%) | 95(83%) | 202(91%) | 148(93%) | 100(92%) | 0.05 |
| GA | 56(9%) | 19(17%) | 17(8%) | 11(7%) | 9(8%) |  |
| AA | 2(.33%) | 0(0%) | 2(1%) | 0(0%) | 0(0%) |  |
| rs3093664 |  |  |  |  |  |  |
| A | 1096 (95%) | 205 (95%) | 425 (96%) | 263 (95%) | 203 (95%) | 0.91 |
| G | 56 (5%) | 11 (5%) | 19 (4%) | 15 (5%) | 11 (5%) |  |
| AA | 524 (91%) | 97 (90%) | 204 (92%) | 125 (90%) | 98 (91%) | 0.63 |
| AG | 48 (8%) | 11 (10%) | 17 (8%) | 13 (9%) | 7 (7%) |  |
| GG | 4 (1%) | 0 | 1 (0.5%) | 1 (1%) | 2 (2%) |  |
| IL-6 |  |  |  |  |  |  |
| rs1800796  (-572 G/C) |  |  |  |  |  |  |
| G | 725(62%) | 164(68%) | 271(62%) | 173(61%) | 117(55%) | **0.04** |
| C | 445(38%) | 76(32%) | 163(38%) | 111(39%) | 95(45%) |  |
| GG | 231(39%) | 56(47%) | 92(42%) | 55(39%) | 28(26%) | **0.02** |
| GC | 263(45%) | 52(43%) | 87(40%) | 63(44%) | 61(58%) |  |
| CC | 91(16%) | 12(10%) | 38(18%) | 24(17%) | 17(16%) |  |
| rs10499563  (-6331 T/C) |  |  |  |  |  |  |
| T | 973(83%) | 196(81%) | 360(83%) | 229(81%) | 188(88%) | 0.15 |
| C | 203(17%) | 46(19%) | 76(17%) | 55(19%) | 26(12%) |  |
| TT | 424(72%) | 84(69%) | 163(75%) | 93(65%) | 84(78%) | **0.007** |
| TC | 125(21%) | 28(23%) | 34(16%) | 43(30%) | 20(19%) |  |
| CC | 39(7%) | 9(7%) | 21(10%) | 6(4%) | 3(3%) |  |
| IL-8 |  |  |  |  |  |  |
| rs2227307 |  |  |  |  |  |  |
| T | 815 (71%) | 151 (70%) | 317 (72%) | 199 (70%) | 148 (70%) | 0.88 |
| G | 311 (28%) | 67 (31%) | 123 (28%) | 85 (30%) | 62 (29%) |  |
| TT | 291(50%) | 51(47%) | 118(54%) | 68(48%) | 54(51%) | 0.74 |
| TG | 234(41%) | 49(45%) | 81(37%) | 63(44%) | 41(39%) |  |
| GG | 52(9%) | 9(8%) | 21(10%) | 11(10%) | 11(10%) |  |
| IL-10 |  |  |  |  |  |  |
| rs1800871  (-819 C/T) |  |  |  |  |  |  |
| C | 666(58%) | 131(68%) | 256(59%) | 158(51%) | 121(58%) | **0.002** |
| T | 484(42%) | 63(32%) | 176(41%) | 156(49%) | 89(42%) |  |
| CC | 195(34%) | 48(50%) | 73(34%) | 42(27%) | 32(31%) | **0.001** |
| CT | 276(48%) | 35(36%) | 110(51%) | 74(47%) | 57(54%) |  |
| TT | 104(18%) | 14(14%) | 33(15%) | 41(26%) | 16(15%) |  |
| rs1800872  (-592 C/A) |  |  |  |  |  |  |
| C | 677(57%) | 141(67%) | 253(58%) | 162(51%) | 121(56%) | **0.005** |
| A | 503(43%) | 71(33%) | 181(42%) | 156(49%) | 95(44%) |  |
| CC | 198(34%) | 48(49%) | 73(34%) | 42(27%) | 32(31%) | **0.001** |
| CA | 276(48%) | 35(36%) | 110(51%) | 74(47%) | 57(54%) |  |
| AA | 104(18%) | 14(14%) | 33(15%) | 41(26%) | 16(15%) |  |
| CXCL6 |  |  |  |  |  |  |
| rs4279174 |  |  |  |  |  |  |
| G | 914 (78%) | 179 (75%) | 342 (80%) | 229 (76%) | 164 (76%) | 0.47 |
| C | 260 (22%) | 59 (25%) | 86 (20%) | 63 (22%) | 52 (24%) |  |
| GG | 364(62%) | 67(56%) | 143(67%) | 90(62%) | 64(59%) | 0.30 |
| GC | 190(32%) | 47(39%) | 56(26%) | 49(34%) | 38(35%) |  |
| CC | 35(6%) | 6(5%) | 15(7%) | 7(5%) | 35(6%) |  |
| CCL5 |  |  |  |  |  |  |
| rs2107538 |  |  |  |  |  |  |
| C | 784 (68%) | 148 (73%) | 289 (66%) | 193 (66%) | 154 (71%) | 0.25 |
| T | 364 (32%) | 56 (27%) | 147 (34%) | 99 (34%) | 62 (29%) |  |
| CC | 270(47%) | 50(49%) | 94(43%) | 68(47%) | 58(53%) | 0.10 |
| CT | 246(43%) | 48(47%) | 101(46%) | 57(39%) | 40(37%) |  |
| TT | 59(10%) | 4(4%) | 23(11%) | 21(14%) | 11(10%) |  |
| rs3817656 |  |  |  |  |  |  |
| A | 817 (72%) | 179 (80%) | 282 (70%) | 194 (68%) | 162 (76%) | **0.009** |
| G | 311 (28%) | 45 (20%) | 124 (30%) | 90 (32%) | 52 (24%) |  |
| AA | 306(54%) | 68(60%) | 105(52%) | 72(51%) | 61(56%) | **0.004** |
| AG | 208(37%) | 44(39%) | 72(36%) | 50(35%) | 42(39%) |  |
| AA | 52(9%) | 1(1%) | 26(13%) | 20(14%) | 5(5%) |  |

^#^Chi-square test, values in bold denotes statistical significance

**Table S2 Association of the Polymorphisms of *TNFα* (rs1800610, rs1800629, rs3093664), *IL-6* (rs1800796, rs10499563), *IL-8* (rs2227307), *IL-10* (rs1800872 and rs1800071), *CXCL6* (rs4279174) and *CCL5* (rs2107538, rs3817656) genes and COVID-19 outcomes**

| **Polymorphisms** | **Moderate** | | | **Severe** | | | **Dead** | | |
| --- | --- | --- | --- | --- | --- | --- | --- | --- | --- |
|  | **OR^#^** | **95% CI** | **P value** | **OR^#^** | **95% CI** | **P value** | **OR^#^** | **95% CI** | **P value** |
| **TNF-a** | | | | | | | | | |
| rs1800610  (+489 G/A) |  |  |  |  |  |  |  |  |  |
| G | Reference |  |  | Reference |  |  | Reference |  |  |
| A | **1.50** | **1.01-2.24** | **0.04** | 1.11 | 0.69-1.79 | 0.65 | 1.30 | 0.74-2.28 | 0.35 |
| GG^c^ | Reference |  |  | Reference |  |  | Reference |  |  |
| GA^c^ | 1.72 | 1.01-2.96 | 0.05 | 1.16 | 0.62-2.17 | 0.65 | 1.11 | 0.52-2.37 | 0.79 |
| AA^c^ | 1.68 | .067-4.08 | 0.25 | 1.13 | 0.37-3.43 | 0.83 | 1.70 | 0.51-5.67 | 0.39 |
| GA+AA^d^ | **1.72** | **1.04-2.84** | **0.04** | 1.15 | 0.64-2.08 | 0.64 | 1.22 | 0.61-2.47 | 0.57 |
| AA^r^ | 1.36 | 0.58-3.21 | 0.48 | 1.08 | 0.36-3.20 | 0.89 | 1.64 | 0.51-5.31 | 0.41 |
|  |  |  |  |  |  |  |  |  |  |
| rs1800629  (-308 G/A) |  |  |  |  |  |  |  |  |  |
| G | Reference |  |  | Reference |  |  | Reference |  |  |
| A | **0.45** | **0.22-0.94** | **0.03** | **0.34** | **0.14-0.83** | **0.02** | 0.52 | 0.20-1.48 | 0.22 |
|  |  |  |  |  |  |  |  |  |  |
| rs3093664 |  |  |  |  |  |  |  |  |  |
| A | Reference |  |  | Reference |  |  | Reference |  |  |
| G | 0.87 | 0.37-2.06 | 0.76 | 0.99 | 0.38-2.59 | 0.99 | 1.06 | 0.35-3.23 | 0.91 |
|  |  |  |  |  |  |  |  |  |  |
|  |  |  |  | **IL-6** |  |  |  |  |  |
| rs1800796  (-572 G/C) |  |  |  |  |  |  |  |  |  |
| G | Reference |  |  | Reference |  |  | Reference |  |  |
| C | 1.35 | 0.92-1.97 | 0.12 | **1.64** | **1.05-2.57** | **0.03** | 1.34 | 0.77-2.34 | 0.3 |
| GG^c^ | Reference |  |  | Reference |  |  | Reference |  |  |
| GC^c^ | 1.03 | 0.60-1.79 | 0.89 | 1.34 | 0.68-2.67 | 0.38 | 1.76 | 0.76-4.08 | 0.18 |
| CC^c^ | 1.87 | 0.83-4.21 | 0.12 | **2.83** | **1.11-7.26** | **0.03** | 1.53 | 0.45-5.18 | 0.48 |
| GC+CC^d^ | 1.20 | 0.72-2.00 | 0.48 | 1.64 | 0.87-3.07 | 0.12 | 1.71 | 0.77-3.77 | 0.18 |
| CC^r^ | 1.84 | 0.86-3.95 | 0.12 | **2.44** | **1.01-5.87** | **0.04** | 1.13 | 0.36-3.53 | 0.83 |
|  |  |  |  |  |  |  |  |  |  |
| rs10499563  (-6331 T/C) |  |  |  |  |  |  |  |  |  |
| T | Reference |  |  | Reference |  |  | Reference |  |  |
| C | 1.04 | 0.65-1.66 | 0.84 | 1.25 | 0.72-2.18 | 0.41 | 0.74 | 0.34-1.62 | 0.46 |
| TT^c^ | Reference |  |  | Reference |  |  | Reference |  |  |
| TC^c^ | 0.69 | 0.36-1.33 | 0.27 | 1.87 | 0.91-3.86 | 0.09 | 0.78 | 0.28-2.13 | 0.63 |
| CC^c^ | 1.58 | 0.63-3.93 | 0.38 | 0.73 | 0.19-2.79 | 0.65 | 0.65 | 0.11-3.64 | 0.62 |
| TC+CC^d^ | 0.91 | 0.52-1.59 | 0.74 | 1.56 | 0.80-3.04 | 0.18 | 0.74 | 0.30-1.86 | 0.53 |
| CC^r^ | 1.69 | 0.68-4.18 | 0.25 | 0.62 | 0.17-2.31 | 0.48 | 0.68 | 0.12-3.78 | 0.66 |
|  |  |  |  |  |  |  |  |  |  |
|  |  |  |  | **IL-8** |  |  |  |  |  |
| rs2227307 |  |  |  |  |  |  |  |  |  |
| T | Reference |  |  | Reference |  |  | Reference |  |  |
| G | 0.91 | 0.61-1.34 | 0.63 | 0.82 | 0.52-1.31 | 0.42 | 0.78 | 0.45-1.34 | 0.37 |
| TT^c^ | Reference |  |  | Reference |  |  | Reference |  |  |
| TG^c^ | 0.71 | 0.41-1.22 | 0.22 | 0.86 | 0.46-1.60 | 0.63 | 0.63 | 0.29-1.34 | 0.24 |
| GG^c^ | 1.11 | 0.44-2.83 | 0.81 | 0.61 | 0.19-1.96 | 0.41 | 0.84 | 0.24-2.94 | 0.79 |
| TG+GG^d^ | 0.77 | 0.47-1.29 | 0.32 | 0.82 | 0.45-1.49 | 0.51 | 0.67 | 0.33-1.36 | 0.27 |
| GG^r^ | 1.30 | 0.53-3.19 | 0.57 | 0.66 | 0.21-2.03 | 0.47 | 1.02 | 0.31-3.41 | 0.97 |
|  |  |  |  |  |  |  |  |  |  |
|  |  |  |  | **IL-10** |  |  |  |  |  |
| rs1800871  (-819 C/T) |  |  |  |  |  |  |  |  |  |
| C | Reference |  |  | Reference |  |  | Reference |  |  |
| T | 1.24 | 0.83-1.85 | 0.28 | **1.94** | **1.24-3.04** | **0.004** | 1.49 | 0.84-2.63 | 0.16 |
| CC^c^ | Reference |  |  | Reference |  |  | Reference |  |  |
| CT^c^ | 1.78 | 0.98-3.24 | 0.06 | 2.01 | 0.98-4.09 | 0.05 | **3.7** | **1.47-9.19** | **0.005** |
| TT^c^ | 1.24 | 0.56-2.78 | 0.06 | **3.00** | **1.28-7.00** | **0.01** | 1.41 | 0.42-4.72 | 0.57 |
| CT+TT^d^ | 1.62 | 0.94-2.82 | 0.08 | **2.33** | **1.21-4.44** | **0.01** | **2.85** | **1.22-6.61** | **0.02** |
| TT^r^ | 0.93 | 0.43-1.94 | 0.83 | 2.09 | 0.97-4.48 | 0.06 | 0.71 | 0.23-2.08 | 0.53 |
|  |  |  |  |  |  |  |  |  |  |
| rs1800872  (-592 C/A) |  |  |  |  |  |  |  |  |  |
| C | Reference |  |  | Reference |  |  | Reference |  |  |
| A | 1.23 | 0.84-1.82 | 0.27 | **1.87** | **1.21-2.89** | **0.004** | **1.73** | **1.01-2.9** | **0.04** |
| CC^c^ | Reference |  |  | Reference |  |  | Reference |  |  |
| CA^c^ | 1.64 | 0.92-2.93 | 0.09 | 1.83 | 0.92-3.64 | 0.08 | **3.17** | **1.32-7.58** | **0.009** |
| AA^c^ | 1.34 | 0.62-2.91 | 0.45 | **2.89** | **1.26-6.60** | **0.01** | 2.31 | 0.74-7.15 | 0.14 |
| CA+AA^d^ | 1.56 | 0.91-2.66 | 0.11 | **2.16** | **1.15-4.03** | **0.02** | **2.91** | **1.28-6.60** | **0.01** |
| AA^r^ | 1.03 | 0.51-2.11 | 0.92 | **2.11** | **1.01-4.44** | **0.04** | 1.19 | 0.44-3.22 | 0.73 |
| Haplotypes |  |  |  |  |  |  |  |  |  |
| CC | Reference |  |  | Reference |  |  | Reference |  |  |
| TA | 1.26 | 0.84-1.89 | 0.25 | **1.97** | **1.25-3.08** | **0.003** | 1.54 | 0.87-2.7 | 0.14 |
|  |  |  |  | **CXCL6** |  |  |  |  |  |
| rs4279174 |  |  |  |  |  |  |  |  |  |
| G | Reference |  |  | Reference |  |  | Reference |  |  |
| C | 0.78 | 0.52-1.19 | 0.26 | 0.72 | 0.44-1.15 | 0.17 | 0.82 | 0.46-1.45 | 0.50 |
| GG^c^ | Reference |  |  | Reference |  |  | Reference |  |  |
| GC^c^ | 0.58 | 0.33-0.99 | 0.05 | 0.65 | 0.35-1.19 | 0.17 | 0.65 | 0.31-1.33 | 0.24 |
| CC^c^ | 1.28 | 0.43-3.79 | 0.66 | 0.66 | 0.17-2.60 | 0.55 | 1.12 | 0.25-4.98 | 0.88 |
| GC+CC^d^ | 0.65 | 0.39-1.08 | 0.10 | 0.65 | 0.36-1.17 | 0.15 | 0.70 | 0.35-1.40 | 0.31 |
| CC^r^ | 1.55 | 0.53-4.53 | 0.42 | 0.77 | 0.20-2.97 | 0.71 | 1.30 | 0.30-5.66 | 0.73 |
|  |  |  |  |  |  |  |  |  |  |
|  |  |  |  | **CCL5** |  |  |  |  |  |
| rs2107538 |  |  |  |  |  |  |  |  |  |
| C | Reference |  |  | Reference |  |  | Reference |  |  |
| T | 1.31 | 0.87-1.95 | 0.19 | 1.22 | 0.76-1.94 | 0.39 | 1.36 | 0.78-2.36 | 0.27 |
| CC^c^ | Reference |  |  | Reference |  |  | Reference |  |  |
| CT^c^ | 0.94 | 0.54-1.62 | 0.83 | 0.73 | 0.39-1.39 | 0.35 | 0.74 | 0.34-1.60 | 0.45 |
| TT^c^ | **3.39** | **1.04-10.9** | **0.04** | 3.27 | 0.92-11.58 | 0.06 | **4.83** | **1.14-20.39** | **0.03** |
| CT+TT^d^ | 1.13 | 0.67-1.90 | 0.66 | 0.95 | 0.52-1.73 | 0.86 | 1.01 | 0.49-2.07 | 0.98 |
| TT^r^ | **3.50** | **1.11-10.95** | **0.03** | **3.75** | **1.10-12.84** | **0.04** | **5.48** | **1.35-22.37** | **0.02** |
|  |  |  |  |  |  |  |  |  |  |
| rs3817656 |  |  |  |  |  |  |  |  |  |
| A | Reference |  |  | Reference |  |  | Reference |  |  |
| G | **1.66** | **1.08-2.54** | **0.02** | **1.64** | **1.02-2.65** | **0.04** | 1.50 | 0.84-2.67 | 0.16 |
| AA^c^ | Reference |  |  | Reference |  |  | Reference |  |  |
| AG^c^ | 0.87 | 0.51-1.50 | 0.62 | 0.82 | 0.43-1.54 | 0.55 | 1.17 | 0.57-2.43 | 0.66 |
| GG^c^ | **14.76** | **1.91-113.7** | **0.01** | **15.63** | **1.92-127.0** | **0.01** | 11.29 | 1.00-127.2 | 0.50 |
| AG+GG^d^ | 1.22 | 0.73-2.04 | 0.45 | 1.21 | 0.67-2.18 | 0.52 | 1.36 | 0.67-2.75 | 0.39 |
| GG^r^ | **15.52** | **2.04-118.3** | **0.008** | **16.74** | **2.09-134.3** | **0.008** | 10.57 | 0.96-116.8 | 0.05 |
| Haplotypes |  |  |  |  |  |  |  |  |  |
| CA | Reference |  |  | Reference |  |  | Reference |  |  |
| TG | 1.39 | 0.87-2.20 | 0.17 | 1.31 | 0.76-2.24 | 0.33 | 1.35 | 0.68-2.67 | 0.38 |

**^#^**Adjusted by age, sex, hypertension, type 2 diabetes and obesity. Inheritance models= c: codominant, d: dominant, r: recessive. Text in bold denotes statistical significance.

**Table S3 Cytokines concentrations between ancestral and minnor alleles of the polymorphisms of *TNFα* (rs1800610, rs1800629, rs3093664), *IL-6* (rs1800796, rs10499563), *IL-8* (rs2227307), *IL-10* (rs1800872 and rs1800071), *CXCL6* (rs4279174) and *CCL5* (rs2107538, rs3817656) genes**

|  | Total | Ancestral allele | Variant allele | P  value |
| --- | --- | --- | --- | --- |
| ***TNF α*** | | | | |
| **rs1800610**  **(+489 G/A)** | **Total** | **G** | **A** | **P**  **value** |
| TNFα pg/mL | 59.2(20.3-183.1) | 54.5(601.9-2141.2) | 79.9(21.8-256.6) | 0.13 |
| IFN α pg/mL | 17.8(10.8-32.9) | 17.6(10.6-31.4) | 18.9(11.8-45.4) | 0.09 |
| IFN γ pg/mL | 51.6(38.5-89.9) | 51.2(37.4-88.9) | 54.2(41.5-93.1) | 0.27 |
| IL-1Ra pg/mL | 215.3(51.3-405.8) | 204.3(45.6-407.2) | 237.6(79.6-380.9) | 0.37 |
| IL-2 pg/mL | 26.7(16.7-78.7) | 26.1(16.2-70.9) | 27.3(18.4-96.3) | 0.23 |
| IL-6 pg/mL | 108.3(31.8-298.5) | 106.5(30.6-262.2) | 126.9(43.4-357.4) | 0.19 |
| IL-7 pg/mL | 125(73.7-404.8) | 123.2(73.7-356.6) | 133(70.6-530.4) | 0.34 |
| IL-10 pg/mL | 39.9(20.3-101.5) | 159.4(23.2-347.9) | 196.2(31.4-375.8) | 0.05 |
| CCL2 pg/mL | 1160.3(356.9-2297.7) | 1115.9(338.2-2255.7) | 1280.1(436.3-2669.6) | 0.22 |
| CCL3 pg/mL | 169.9(25.5-360.7) | 159.4(23.2-347.9) | 196.2(31.3-375.8) | 0.52 |
| CXCL8 pg/mL | 179.3(54.2-334.2) | 175.1(52.3-309.8) | 204.6(73.8-382.2) | 0.19 |
| CXCL10 pg/mL | 1299.9(651.6-2206.8) | 1230.6(601.9-2141.2) | 1503.9(686.6-2471.1) | 0.06 |
| GCSF pg/mL | 29.6(17.3-54.8) | 28.4(17.6-52.5) | 37.1(16.4-65.3) | 0.17 |
|  |  |  |  |  |
| **rs1800629**  **(-308 G/A)** | **Total** | **G** | **A** | **P**  **value** |
| TNFα pg/mL | 62.9(20.5-190.5) | 63.7(20.3-191.6) | 43.9(23.7-137.3) | 0.83 |
| IFN α pg/mL | 17.9(10.8-33.7) | 17.9(10.6-33.8) | 17.1(12.4-23.3) | 0.98 |
| IFN γ pg/mL | 51.7(38.8-91.3) | 52.9(39.0-91.3) | 44.9(35.4-89.9) | 0.23 |
| IL-1Ra pg/mL | 217.2(61.3-408.4) | 224.7(64.0-424.1) | 61.0(24.9-220.2) | **0.003** |
| IL-2 pg/mL | 27.0(16.7-79.3) | 27.2(16.9-79.7) | 18.8(13.9-34.7) | 0.11 |
| IL-6 pg/mL | 109.9(33.3-302.7) | 114.3(35.4-308.7) | 43.8(22.9-143.5) | **0.01** |
| IL-7 pg/mL | 127.8(73.9-428.2) | 129.0(75.2-433.9) | 99.3(52.7-164.9) | 0.11 |
| IL-10 pg/mL | 40.4(20.1-101.5) | 41.1(20.3-101.8) | 23.5(18.2-49.9) | 0.05 |
| CCL2 pg/mL | 1171.6(376.9-2361.5) | 1191.7(378.2-2374.9) | 494.9(195.3-1674.5) | **0.02** |
| CCL3 pg/mL | 169.9(27.2-368.3) | 177.8(30.8-368.3) | 85.6(0.5-441.9) | 0.53 |
| CXCL8 pg/mL | 185.5(57.4-338.2) | 188.5(60.3-341.1) | 153.6(24.8-244.4) | 0.18 |
| CXCL10 pg/mL | 1299.9(651.6-2153.2) | 1317.4(660.9-2230.1) | 741.8(333.3-1400.5) | **0.002** |
| GCSF pg/mL | 28.9(16.7-55.0) | 29.6(16.7-57.7) | 24.8(17.6-41.6) | 0.33 |
|  |  |  |  |  |
| **rs3093664** | **Total** | **A** | **G** | **P value** |
| TNFα pg/mL | 59.2(20.3-183.1) | 54.5(19.5-173.7) | 79.9(21.8-256.6) | 0.05 |
| IFN α pg/mL | 17.6(10.6-32.3) | 17.1(10.45-32.6) | 22.5(13.5-31.7) | 0.27 |
| IFN γ pg/mL | 51.2(37.9-88.9) | 51.1(37.6-89.4) | 55.3(41.8-88.9) | 0.51 |
| IL-1Ra pg/mL | 215.3(51.3-405.8) | 204.3(45.5-407.1) | 237.6(79.6-380.9) | 0.84 |
| IL-2 pg/mL | 25.8(16.5-75.5) | 25.7(16.4-73.6) | 34.7(18.5-102.1) | 0.31 |
| IL-6 pg/mL | 111.2(30.6-286.1) | 112.4(30.6-286.2) | 96.3(52.3-197.6) | 0.84 |
| IL-7 pg/mL | 117(70.6-401.9) | 116.4(69.5-397.1) | 181.7(93.1-501.8) | 0.13 |
| IL-10 pg/mL | 39.9(20.3-101.5) | 37.5(20.2-93.5) | 51.2(23.8-131.9) | 0.15 |
| CCL2 pg/mL | 1160.3(352.8-2339.6) | 1153.8(356.9-2350.5) | 1714.5(292.3-2242.6) | 0.97 |
| CCL3 pg/mL | 169.9(25.5-360.7) | 159.3(23.1-347.9) | 196.2(31.3-375.8) | 0.32 |
| CXCL8 pg/mL | 179.3(54.1-334.2) | 175.1(52.3-309.8) | 204.6(73.8-382.1) | 0.41 |
| CXCL10 pg/mL | 1299.9(651.6-2206.7) | 1230.6(601.8-2141.2) | 1503.9(686.6-2471.1) | 0.94 |
| GCSF pg/mL | 28.9(16.5-53.3) | 28.4(10.5-53.8) | 35.2(19.7-50.2) | 0.65 |
|  |  |  |  |  |
| ***IL-6*** | | | | |
| **rs1800796**  **(-572 G/C)** | **Total** | **G** | **C** | **P value** |
| TNFα pg/mL | 54.5(18.6-173.7) | 52.2(20.3-173.7) | 57.4(16.2-173.7) | 0.51 |
| IFN α pg/mL | 17.6(10.7-32.3) | 18.2(11.4-32.9) | 15.9(9.4-30.9) | 0.08 |
| IFN γ pg/mL | 51.1(37.8-87.4) | 51.7(37.8-88.9) | 48.7(37.4-84.9) | 0.57 |
| IL-1Ra pg/mL | 214.5(44.8-405.8) | 195.9(44.1-367.3) | 228.9(64.1-458.7) | 0.13 |
| IL-2 pg/mL | 25.6(16.2-73.6) | 25.5(15.9-75.5) | 25.8(16.9-72.8) | 0.79 |
| IL-6 pg/mL | 108.3(29.7-286.1) | 106.4(30.62-286.02) | 119(28.9-286.2) | 0.89 |
| IL-7 pg/mL | 116.2(69.5-395.8) | 125(73.7-404.8) | 11.3(62.5-352.8) | 0.21 |
| IL-10 pg/mL | 37.5(20.1-98.8) | 40.6(20.5-101.9) | 34.8(19.5-94.1) | 0.18 |
| CCL2 pg/mL | 1157.1(342.9-2283-.9) | 1078.9(334.4-2297.7) | 1239.4(361.1-2242.6) | 0.68 |
| CCL3 pg/mL | 166.9(24.9-368.3) | 155.8(24.9-348.9) | 186.7(24.3-386.8) | 0.63 |
| CXCL8 pg/mL | 175.3(50.8-312.5) | 178.9(51.3-324.8) | 173.2(49.5-309.7) | 0.81 |
| CXCL10 pg/mL | 1230.6(629.9-2151.1) | 1237.3(665.9-2151.1) | 1223.8(529.3-2131.3) | 0.54 |
| GCSF pg/mL | 28.7(16.5-54.5) | 29.6(18.2-54.5) | 26.3(15.4-53.1) | 0.38 |
|  |  |  |  |  |
| **rs10499563**  **(-6331 T/C)** | **Total** | **T** | **C** | **P value** |
| TNFα pg/mL | 57.4(19.5-173.7) | 53.5(18.1-171.8) | 78.8(23.5-209.5) | 0.14 |
| IFN α pg/mL | 17.9(10.8-33.7) | 17.5(10.1-31.4) | 20.6(11.4-50.4) | **0.04** |
| IFN γ pg/mL | 51.2(37.8-88.8) | 49.2(37.6-85.8) | 60.6(40.8-102.1) | 0.07 |
| IL-1Ra pg/mL | 214.9(49.6-405.8) | 200.8(46.2-386.6) | 264.4(55.4-488.8) | 0.12 |
| IL-2 pg/mL | 25.7(16.5-73.6) | 25.5(16.1-73.2) | 33.2(18.4-79.7) | 0.09 |
| IL-6 pg/mL | 109.9(30.6-302.7) | 101.3(29.5-262.2) | 184.2(44.8-372.7) | **0.03** |
| IL-7 pg/mL | 116.5(70.6-398.4) | 115.6(68.4-378.2) | 163.9(83.3-487.4) | 0.05 |
| IL-10 pg/mL | 38.9(20.2-100.2) | 36.9(20.1-95.5) | 52.5(24.3-103.9) | 0.07 |
| CCL2 pg/mL | 1160.3(342.9-2361.4) | 1092.2(338.2-2242.6) | 1665.4(436.3-2736.1) | **0.04** |
| CCL3 pg/mL | 169.2(24.9-360.7) | 154.8(23.1-356.7) | 240.8(77.3-375.8) | 0.09 |
| CXCL8 pg/mL | 175.3(52.3-312.5) | 173.9(49.2-310.7) | 204.9(78.9-390.8) | 0.10 |
| CXCL10 pg/mL | 1238.2(629.9-2206.7) | 1230.6(601.9-2179.9) | 1424.5(688.3-2652.5) | 0.24 |
| GCSF pg/mL | 28.9(16.9-54.5) | 27.9(16.6-53.2) | 33.45(19.5-60.6) | 0.14 |
| ***IL-8*** | | | | |
| **rs2227307** | **Total** | **T** | **G** | **P**  **value** |
| TNFα pg/mL | 58.1(19.5-175.8) | 51.4(18.3-163.9) | 88.9(22.7-226.4) | 0.05 |
| IFN α pg/mL | 17.6(10.6-32.9) | 16.7(10.3-30.9) | 18.3(10.8-43.1) | 0.20 |
| IFN γ pg/mL | 51.1(37.9-88.9) | 48.9(38.3-84.2) | 58.4(35.6-96.0) | 0.35 |
| IL-1Ra pg/mL | 215.4(51.1-408.4) | 215.1(40.9-408.4) | 215.9(85.4-414.9) | 0.46 |
| IL-2 pg/mL | 26.1(16.7-75.5) | 25.6(16.1-70.8) | 30.9(18.6-80.2) | 0.07 |
| IL-6 pg/mL | 111.2(31.8-302.7) | 108.4(29.9-304.4) | 120.1(44.2-290.2) | 0.62 |
| IL-7 pg/mL | 123.2(73.7-401.9) | 116.0(66.4-371.2) | 135.1(80.8-480.3) | 0.09 |
| IL-10 pg/mL | 38.1(20.1-100.2) | 36.7(19.9-92.8) | 44.8(22.1-105.1) | 0.17 |
| CCL2 pg/mL | 1172.3(356.1-2374.9) | 1126.4(344.2-2357.3) | 1320.8(426.5-2644.5) | 0.21 |
| CCL3 pg/mL | 162.4(24.9-368.3) | 153.8(17.9-352.8) | 214.3(34.6-401.1) | 0.15 |
| CXCL8 pg/mL | 177.0(55.2-324.7) | 173.9(49.8-311.9) | 189(78.9-336.2) | 0.22 |
| CXCL10 pg/mL | 1282.9(657.2-2151.1) | 1187.6(605.3-2122.9) | 1430.2(700.4-2463.8) | 0.09 |
| GCSF pg/mL | 28.8(16.5-54.5) | 29.6(16.5-54.6) | 28.1(16.6-54.5) | 0.59 |
| ***IL-10*** | | | | |
| **rs1800871**  **(-819 C/T)** | **Total** | **C** | **T** | **P**  **value** |
| TNFα pg/mL | 66.1(20.7-191.7) | 66.2(19.5-205.1) | 66(21.8-173.7) | 0.70 |
| IFN α pg/mL | 17.9(10.8-34.3) | 17.7(10.9-36.8) | 18.1(9.7-33.3) | 0.39 |
| IFN γ pg/mL | 53.5(39-92.4) | 51.6(38.5-95.8) | 54.4(40.2-85.8) | 0.96 |
| IL-1Ra pg/mL | 226.6(69.2-433.2) | 215.7(53.3-413.2) | 253.3(101.7-437.2) | 0.07 |
| IL-2 pg/mL | 27.4(16.9-81.8) | 27.8(16.6-87.5) | 27.3(17.5-79.3) | 0.99 |
| IL-6 pg/mL | 126.2(36.3-316.8) | 114.3(30.6-332.6) | 126.9(4.8-304.4) | 0.39 |
| IL-7 pg/mL | 130.2(76.4-459.6) | 132(76.1-488) | 128.5(76.4-398.4) | 0.64 |
| IL-10 pg/mL | 40.6(20.3-95.3) | 40.6(19.9-109.3) | 39.04(20.3-95.3) | 0.35 |
| CCL2 pg/mL | 1258.3(436.3-2506.2) | 1172.9(342.9-2506.2) | 1324.8(525.9-2644.4) | 0.14 |
| CCL3 pg/mL | 186.7(32.4-375.8) | 169.1(20.5-362.1) | 217.9(51.4-387.2) | 0.13 |
| CXCL8 pg/mL | 193.6(66.7-343.1) | 189.9(57.4-363.9) | 197.2(78.9-311.5) | 0.77 |
| CXCL10 pg/mL | 1322.5(667.1-2206.8) | 1360.9(667.1-2213.7) | 1273.8(666.3-2153.2) | 0.97 |
| GCSF pg/mL | 29.6(16.9-81.8) | 29.9(18-63.6) | 28.4(16.4-51.4) | 0.28 |
|  |  |  |  |  |
| **rs1800872**  **(-592 C/A)** | **Total** | **C** | **A** | **P**  **value** |
| TNFα pg/mL | 63.7(20.5-191.6) | 63.7(18.6-205.6) | 63.7(21.8-173.7) | 0.71 |
| IFN α pg/mL | 17.9(10.8-34.3) | 17.6(10.9-37.7) | 18.1(9.9-32.3) | 0.40 |
| IFN γ pg/mL | 52.9(39-91.7) | 51.4(38.3-95.5) | 53.7(40.9-84.9) | 0.91 |
| IL-1Ra pg/mL | 224.8(66.7-424.1) | 215.4(55.4-397.2) | 248.8(85.4-434.1) | 0.12 |
| IL-2 pg/mL | 27.3(16.9-81) | 27.5(16.3-85.6) | 27(17-79.3) | 0.97 |
| IL-6 pg/mL | 120.6(35.7-308.7) | 109.9(30.6-329.9) | 126.6(43.6-304.4) | 0.45 |
| IL-7 pg/mL | 128.4(73.9-436.8) | 130.9(75.17-487.9) | 123.2(70.32-395.8) | 0.57 |
| IL-10 pg/mL | 40.6(20.12-102.5) | 40.6(19.9-109.3) | 39.04(20.3-95.2) | 0.32 |
| CCL2 pg/mL | 1214.3(415.1-2470.8) | 1162.1(342.9-2470.8) | 1320.8(506.7-2470.8) | 0.16 |
| CCL3 pg/mL | 180.9(30.8-375.8) | 168.3(17.9-363.5) | 209.7(46.7-386.8) | 0.20 |
| CXCL8 pg/mL | 189.9(62.8-343.1) | 189.9(57-365.1) | 192.9(73.2-309.8) | 0.98 |
| CXCL10 pg/mL | 1329.7(667.1-220.6) | 1348.9(667.1-22020.6) | 1317.2(666.3-2220.6) | 0.94 |
| GCSF pg/mL | 29.6(16.7-58.5) | 29.7(17-64.2) | 28.1(16.4-51.2) | 0.28 |
| **CXCL6** | | | | |
| **rs4279174** | **Total** | **G** | **C** | **P**  **value** |
| TNFα pg/mL | 57.5(19.7-174.7) | 53.5(18.6-172.1) | 72.1(22.9-192.8) | 0.25 |
| IFN α pg/mL | 17.8(10.7-32.6) | 17.2(10.1-31.7) | 18.5(11.7-41.2) | 0.18 |
| IFN γ pg/mL | 50.8(37.8-88.1) | 48.7(37.4-84.6) | 55.8(40.2-95.2) | 0.20 |
| IL-1Ra pg/mL | 215.4(50.4-401.5) | 215.4(49.62-397.2) | 215.4(61.6-405.7) | 0.91 |
| IL-2 pg/mL | 25.9(16.6-77.6) | 25.8(16.2-76.7) | 26.7(18.5-79.3) | 0.45 |
| IL-6 pg/mL | 109.9(31.2-298.5) | 106.8(31.8-304.4) | 119.6(30.6-273.5) | 0.93 |
| IL-7 pg/mL | 119.2(72.1-403.3) | 116.4(65.5-404.8) | 132.6(82.9-352.8) | 0.30 |
| IL-10 pg/mL | 39.5(20.3-101.3) | 38.6(20.2-101.5) | 40.4(23.1-97.4) | 0.74 |
| CCL2 pg/mL | 1161.2(346.6-2350.5) | 1147.6(342.9-2374.9) | 1260.26(361.1-2169.9) | 0.70 |
| CCL3 pg/mL | 166.9(24.89-362.07) | 159.1(23.13-352.82) | 237.71(32.4-372.11) | 0.29 |
| CXCL8 pg/mL | 177.02(52.99-323.64) | 175.46(51.29-338.2) | 178.6(57.7-312.4) | 0.89 |
| CXCL10 pg/mL | 1278.1(647.0-2213.7) | 1237.4(629.9-2151.1) | 1395.8(667.0-2360.9) | 0.23 |
| GCSF pg/mL | 29.2(17.2-54.9) | 29.6(16.9-58.5) | 25.5(17.9-52) | 0.18 |
|  |  |  |  |  |
| ***CCL5*** | | | | |
| **rs2107538** | **Total** | **C** | **T** | **P value** |
| TNFα pg/mL | 58.8(19.5-179.9) | 51.6(19.5-168.7) | 85.7(22.2-205.6) | 0.09 |
| IFN α pg/mL | 17.6(10.62-32.91) | 16.68(10.1-30.85) | 19.68(11.84-35.06) | 0.05 |
| IFN γ pg/mL | 51.1(38.3-90.9) | 48.9(37.8-87.3) | 55.8(40.9-95.5) | 0.16 |
| IL-1Ra pg/mL | 215.7(61.3-424.1) | 201.5(42.2-424.1) | 230.4(95.6-408.4) | 0.11 |
| IL-2 pg/mL | 26.8(16.9-78.7) | 24.3(15.9-67.9) | 33.3(18.5-95.5) | **0.002** |
| IL-6 pg/mL | 122.8(31.8-306.6) | 97.7(29.9-273.6) | 146.7(44.4-358.4) | **0.02** |
| IL-7 pg/mL | 125(73.6-404.8) | 112.5(64.5-337.7) | 167.5(81-517.9) | **0.003** |
| IL-10 pg/mL | 39.5(20.2-101.5) | 36.2(19.8-90.9) | 54.9(24.3-131.9) | **0.02** |
| CCL2 pg/mL | 1191.66(361.49-2452.61) | 1047.31(334.44-2297.7) | 1446.3(665.68-2644.46) | **0.007** |
| CCL3 pg/mL | 166.9(24.9-372.1) | 149.8(17.9-372.1) | 220.5(37.0-380.6) | 0.14 |
| CXCL8 pg/mL | 179.9(57.4-325.1) | 171.3(42.6-286.3) | 206.7(103.3-382.1) | **0.002** |
| CXCL10 pg/mL | 1310.7(666.3-2206.7) | 1182.5(605.3-2117.9) | 1611.5(697.1-2652.8) | **0.02** |
| GCSF pg/mL | 29.2(16.5-54.5) | 27.3(16.3-51.2) | 36.7(20.5-64.2) | **0.02** |
|  |  |  |  |  |
| **rs3817656** | **Total** | **A** | **G** | **P value** |
| TNFα pg/mL | 54.4(18.3-173.7) | 51.2(18.6-168.7) | 75.6(17.4-191.6) | 0.37 |
| IFN α pg/mL | 17.6(10.2-31.9) | 17.0(10.1-30.8) | 19.7(10.79-32.9) | 0.26 |
| IFN γ pg/mL | 48.9(37.4-84.9) | 48.7(37.4-84.2) | 54.4(37.4-93.1) | 0.45 |
| IL-1Ra pg/mL | 215.3(51.1-405.8) | 199.5(42.2-376.0) | 230.4(85.3-408.4) | 0.11 |
| IL-2 pg/mL | 25.7(16.5-70.9) | 24.0(15.5-64.2) | 30.9(17.9-92.2) | **0.008** |
| IL-6 pg/mL | 109.9(31.8-306.6) | 95.7(29.5-254.8) | 137.2(44.0-400.7) | **0.02** |
| IL-7 pg/mL | 116.4(70.3-352.8) | 111.2(64.5-263.8) | 133.35(79.94-484.67) | **0.03** |
| IL-10 pg/mL | 37.6(20.1-97.4) | 35.4(19.8-83.9) | 50.9(23.1-114.9) | 0.11 |
| CCL2 pg/mL | 1162.0(352.8-2374.9) | 1032.9(327.4-2283.8) | 1425.7(665.7-2653.5) | **0.02** |
| CCL3 pg/mL | 164.7(23.1-368.3) | 153.8(7.6-368.3) | 215.1(37.0-352.8) | 0.36 |
| CXCL8 pg/mL | 178.6(52.3-311.5) | 171.3(41.8-284.5) | 200.7(97.0-366.6) | **0.01** |
| CXCL10 pg/mL | 1299.9(642.4-2206.7) | 1182.0(601.8-2124.4) | 1545.3(666.3-2567.9) | 0.14 |
| GCSF pg/mL | 28.2(16.7-54.5) | 26.4(46.4-51.4) | 31.4(18.3-63.0) | 0.12 |
| **IL-10 HAPLOTYPES** | | | | |
| TNFα pg/mL | 67.8(20.8-192.8) | 67.8(19.5-205.6) | 67.8(22.4-179.9) | 0.82 |
| IFN α pg/mL | 18(10.8-35.01) | 17.7(10.9-37.6) | 18.1(9.8-33.3) | 0.45 |
| IFN γ pg/mL | 53.9(39.0-93.1) | 51.7(38.5-96.2) | 54.7(40.9-85.9) | 0.94 |
| IL-1Ra pg/mL | 228.9(70.5-434.1) | 215.9(61.3-420.6) | 255.1(105.3-442.2) | 0.08 |
| IL-2 pg/mL | 27.8(16.9-85.5) | 27.8(16.7-87.5) | 27.4(17.6-81.8) | 0.92 |
| IL-6 pg/mL | 125.7(37.6-307.7) | 114.3(31.8-329.9) | 126.8(45.9-304.4) | 0.39 |
| IL-7 pg/mL | 130.3(76.4-466.3) | 132.9(76.4-488.1) | 130.2(76.4-401.9) | 0.65 |
| IL-10 pg/mL | 41.1(20.1-102.5) | 40.6(19.9-109.3) | 42.8(20.2-95.2) | 0.37 |
| CCL2 pg/mL | 1277.3(439.2-2506.2) | 1172.9(345.5-2506.2) | 1324.8(525.9-2644.5) | 0.14 |
| CCL3 pg/mL | 191.3(33.1-380.6) | 173.4(23.1-363.5) | 221.2(54.9-387.2) | 0.11 |
| CXCL8 pg/mL | 195.6(67.2-344.3) | 190.1(57.7-365.1) | 197.8(78.9-312.4) | 0.75 |
| CXCL10 pg/mL | 1336.8(670.5-220.6) | 1378.3(670.5-2220.6) | 1312.0(670.5-2220.6) | 0.99 |
| GCSF pg/mL | 29.6(16.9-60.03) | 30.01(17.9-64.1) | 28.8(16.4-51.4) | 0.34 |
| **CCL-5 HAPLOTYPES** | | | | |
| TNFα pg/mL | 54.5(18.6-173.7) | 51.2(18.6-168.7) | 75.6(20.8-191.6) | 0.23 |
| IFN α pg/mL | 17.5(9.9-31.8) | 16.1(9.7-27.9) | 19.7(10.8-32.9) | 0.11 |
| IFN γ pg/mL | 48.9(37.4-87.4) | 48.7(37-84.3) | 54.6(37.8-93.1) | 0.31 |
| IL-1Ra pg/mL | 215.4(61.3-434.1) | 201.5(44.8-433.2) | 250.3(101.7-440.5) | 0.07 |
| IL-2 pg/mL | 25.8(16.8-72.8) | 24.2(15.9-64.0) | 32.5(18.3-92.2) | **0.003** |
| IL-6 pg/mL | 120.06(31.9-323.4) | 101.3(29.9-294.3) | 142.7(44.4-400.6) | **0.02** |
| IL-7 pg/mL | 116.4(70.6-360.5) | 111.2(64.5-263.8) | 133.5(79.9-488.1) | **0.01** |
| IL-10 pg/mL | 37.4(19.9-97.4) | 34.9(19.5-78.4) | 50.9(23.1-117.4) | 0.05 |
| CCL2 pg/mL | 1191.7(376.9-2506.2) | 1078.8(342.9-2374.9) | 1446.3(713.8-2796.1) | **0.008** |
| CCL3 pg/mL | 164.7(24.3-380.6) | 153.8(7.6-375.8) | 218.4(37.0-386.8) | 0.22 |
| CXCL8 pg/mL | 179.9(57.4-309.8) | 173.2(48.8-284.5) | 206.7(103.7-366.6) | **0.005** |
| CXCL10 pg/mL | 1309.5(657.2-2131.3) | 1182.1(605.3-2118.4) | 1545.3(666.3-2626.5) | 0.07 |
| GCSF pg/mL | 28.2(16.4-53.3) | 26.4(15.7-51.2) | 33.7(18.1-64.1) | 0.05 |

All data are presented in median (Interquartile Range). P-value obtained from Kruskal-Wallis test. Values in bold denotes statistical significance.
